# A Piezoelectric Resonance Pump Based on a Flexible Support

**DOI:** 10.3390/mi10030169

**Published:** 2019-02-28

**Authors:** Jiantao Wang, Xiaolong Zhao, Xiafei Chen, Haoren Yang

**Affiliations:** 1 Postdoctoral Mobile Station of Mechanical Engineering, Yanshan University, Qinhuangdao 066004, China; 2 School of Vehicle and Energy, Yanshan University, Qinhuangdao 066004, China; 3 School of Mechanical Engineering, Yanshan University, Qinhuangdao 066004, China; xlzhao@stumail.ysu.edu.cn (X.Z.); chenxiafi@163.com (X.C.); yanghaoren9@163.com (H.Y.)

**Keywords:** square piezoelectric vibrator, resonance, piezoelectric diaphragm pump, flexible support, piezoelectric resonance pump

## Abstract

Small volume changes are important factors that restrict the improvement of the performance of a piezoelectric diaphragm pump. In order to increase the volume change of the pump chamber, a square piezoelectric vibrator with a flexible support is proposed in this paper and used as the driving unit of the pump. The pump chamber diaphragm was separated from the driving unit, and the resonance principle was used to amplify the amplitude of the pump diaphragm. After analyzing the working principle of the piezoelectric resonance pump and establishing the motion differential equation of the vibration system, prototypes with different structural parameters were made and tested. The results show that the piezoelectric resonance pump resonated at 236 Hz when pumping air. When the peak-to-peak voltage of the driving power was 220 V, the amplitude of the diaphragm reached a maximum value of 0.43933 mm, and the volume change of the pump was correspondingly improved. When the pump chamber height was 0.25 mm, the output flow rate of pumping water reached a maximum value of 213.5 mL/min. When the chamber height was 0.15 mm, the output pressure reached a maximum value of 85.2 kPa.

## 1. Introduction

The piezoelectric diaphragm pump, which uses a piezoelectric actuator as a drive unit, is a common form of positive displacement pump. This type of pump has the advantages of simple structure, easy miniaturization, zero electromagnetic interference and low noise, so it is widely used in many fields such as biomedicine [1,2,3], fuel cells [4,5,6], cooling systems [7,8,9], and household appliances [10].

A typical piezoelectric diaphragm pump uses a circular piezoelectric vibrator to directly drive fluid. In order to construct a closed volume pump chamber, the vibrator must be sealed to the periphery of the pump body. This installation limits the deformation of the vibrator, making it difficult for the pump chamber to obtain a large volume change. To eliminate the limitations on the structure and installation, researchers have separated the piezoelectric actuator from the diaphragm of the pump chamber to make an indirect drive piezoelectric diaphragm pump [9,11,12]. Meanwhile, the resonance principle is used to amplify the vibration displacement of the piezoelectric actuator to drive the diaphragm—and to increase the volume change of the pump [12]—in order to form a piezoelectric resonance diaphragm pump (hereafter referred to as a piezoelectric resonance pump).

The driving device of the early piezoelectric resonance pump was mainly composed of a piezoelectric stack [5,9,13,14,15]. It had a large driving force and high precision, but the manufacturing process was complex, with the features of high cost, large volume and high replacement cost if damaged. In recent years we have seen an increase in studies of piezoelectric resonance pumps. These studies mainly used piezoelectric vibrators in different shapes, such as circular, annular and rectangular vibrators, as the driving device of the pump.

In 2012, Jilin University developed a gas piezoelectric resonance pump with a large pump chamber compression ratio, using an annular bimorph piezoelectric vibrator as the driving device [11]. In 2014, for the precise transportation of chemical fuel cells, the University of Science and Technology of China developed a piezoelectric resonance pump with a flexible fluid buffer cavity at the outlet and inlet of the pump chamber. The pump used the rectangular piezoelectric vibrator with free ends as its driving device [16] and used the vibration inertia of the vibrator to drive the movement of the diaphragm in the pump chamber. In 2015, the University of Science and Technology of China also designed a piezoelectric resonance pump with double pump chamber units [17]. It consisted of a U-shaped piezoelectric actuator and two diaphragm pump chamber units that were symmetrically arranged. The U-shaped actuator had very good displacement driving capability in the resonant state. In 2016, National Taiwan University developed a split-type piezoelectric resonance pump used in the medical industry [1]. The circular bimorph piezoelectric vibrator drove the diaphragm through the middle strut. The pump chamber unit could be freely disassembled and replaced, reducing the cost of disposals.

Piezoelectric actuators can be divided into several types according to their structures, such as unimorph/bimorph piezoelectric vibrators [10,11], piezoelectric stacks [5], cylindrical piezoelectric actuators [18], cymbal-shaped piezoelectric actuators [19] and so on. These piezoelectric actuators are based on the strain induced in the longitudinal or transverse directions. The unimorph/bimorph piezoelectric vibrator has the characteristics of simple structure, low cost and various forms. Specifically, the annular and the circular piezoelectric vibrators mainly adopt a periphery fixed installation method, which suits a compact piezoelectric resonance pump system [20]. In contrast, the rectangular piezoelectric vibrator is more flexible in installation. For example, it can be fixed at both ends, at one end, or with both free ends. This method is suitable for a piezoelectric resonance pump system with a large displacement output [12]. After analyzing the advantages and characteristics of common piezoelectric vibrators, a square vibrator with a flexible support is proposed in this paper. The vibrator’s substrate adopts a hollow design, with only four corners fixed, to form the flexible support. This structure can reduce the constraint of the support method on the vibration of the square vibrator, thereby improving the vibrator’s performance on the displacement output. In this study, the abovementioned piezoelectric vibrator was used as the driving device to construct the piezoelectric resonance pump. We also analyzed and calculated a dynamic model of the pump’s vibration and conducted experimental research on the prototype.

## 2. Development of the Piezoelectric Resonance Pump

### 2.1. Design Concept

**Figure 1:**
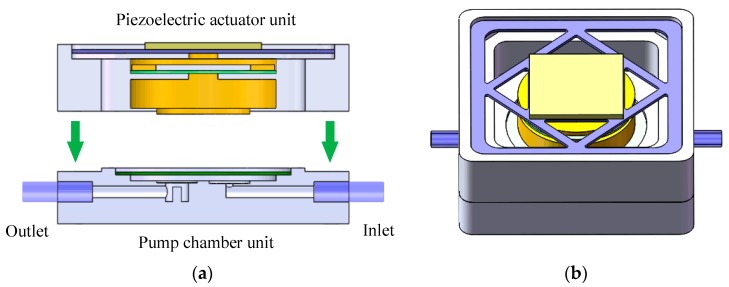
Structure of the piezoelectric resonance pump; (**a**) cross-sectional view; (**b**) assembly view.

Figure 1a shows the overall structure of the piezoelectric resonance pump, which includes a piezoelectric actuator unit and a pump chamber unit. A square piezoelectric vibrator is the core of the actuator unit, and can periodically vibrate when driven by the sine wave signal. Under it is the elastic vibration transfer block, which is used to amplify the vibrator’s amplitude to drive the pump chamber diaphragm. The block is also the stiffness and mass adjusting element of the pump vibration system.

The pump body, processed by polymethyl methacrylate (PMMA), and the circular elastic diaphragm, made from beryllium bronze, were glued together to form an airtight pump chamber unit. The inlet and outlet of the pump employed wheeled check valves. The valve piece was cut from beryllium bronze by ultraviolet laser and bonded to the inlet and outlet seats. Due to the one side limit, the wheeled valve at the inlet only opened to the inside of the pump chamber, and the outlet wheeled valve only to the outside. Figure 1b is the assembly view of the piezoelectric resonance pump.

**Figure 2:**
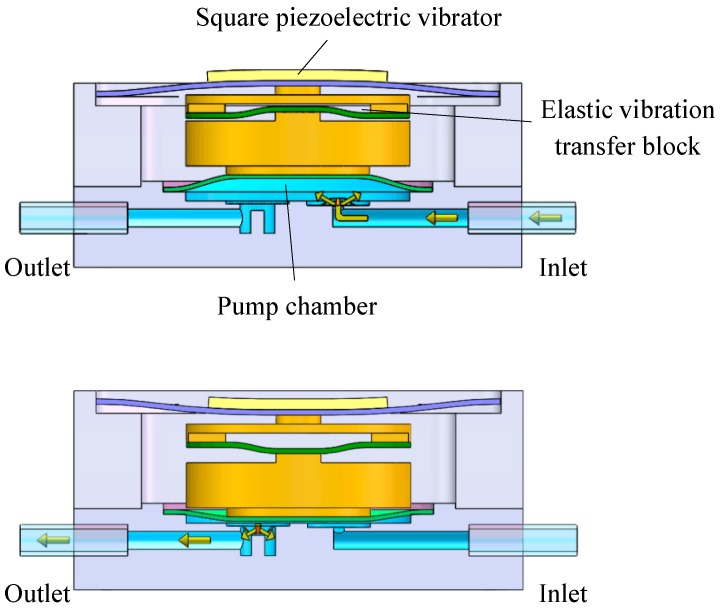
Schematic diagram of pump operation.

Figure 2 is the schematic of the piezoelectric resonance pump when pumping liquid. The square piezoelectric vibrator periodically vibrates when it is driven by the sine wave signal. In resonance, the center amplitude of the vibrator can be amplified by the elastic vibration transfer block. When the vibrator moves upward, the pump chamber diaphragm moves upwards as well, under the driving force of the block. The pressure in the pump chamber is lowered with the inlet valve open and the outlet valve closed, at which point liquid flows into the pump chamber through the inlet. Conversely, when the vibrator moves downward, the pump chamber diaphragm moves downwards under the action of the elastic vibration transfer block. The pressure in the pump chamber increases with the inlet valve closed and the outlet valve open, at which point liquid flows out through the outlet.

### 2.2. Design of the Piezoelectric Vibrator with Flexible Support

**Figure 3:**
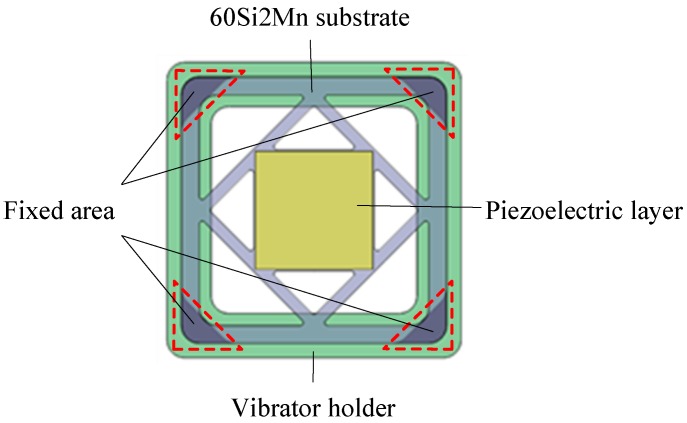
Piezoelectric vibrator based on a flexible support.

Figure 3 is a structural diagram of the square piezoelectric vibrator designed in this paper. It consists of a 60Si2Mn substrate and a square piezoelectric layer, bonded together. The metal substrate adopts a hollow design, and its four corners are fixed on the vibrator holder, thereby forming a structure with flexible support for the square piezoelectric vibrator. This structure can reduce the vibration constraint of the vibrator and concentrate the deformation in the hollow area during the vibration. The metal substrate with flexible support can improve the mechanical properties of the piezoelectric vibrator and protect the piezoelectric layer.

## 3. Dynamic Model

**Figure 4:**
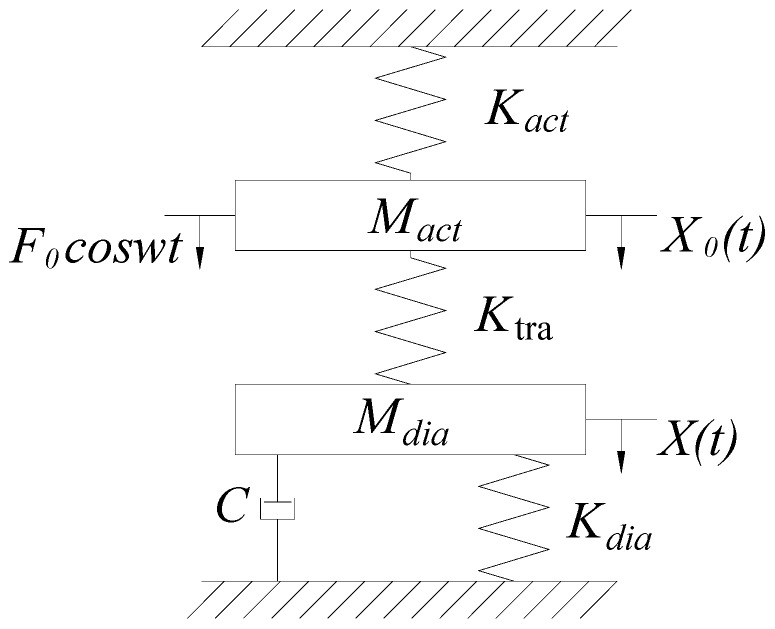
Dynamic model of the piezoelectric resonance pump.

The piezoelectric resonance pump uses system resonance to amplify the amplitude of the piezoelectric vibrator and drive the pump chamber diaphragm to improve the volume change of the pump chamber and optimize the pump’s output performance. The dynamic model of the vibration system is established in this section to study the influence of system parameters on the natural frequency and amplitude amplification coefficient. Figure 4 shows the simplified dynamic model of the piezoelectric resonance pump. *M_act_* is the equivalent mass of the square piezoelectric vibrator; *M_dia_* is the equivalent mass of elastic vibration transfer block and pump chamber diaphragm; *K_act_* is the equivalent stiffness of the square piezoelectric vibrator; *K_tra_* is the equivalent stiffness of the elastic vibration transfer block; and *K_dia_* is the equivalent stiffness of the pump chamber diaphragm. The interaction between the fluid and the flow channel is equivalent to damping *C*, ignoring the material damping of the elastic element in the vibration system.

The square piezoelectric vibrator uses a sine wave driving power, and *w* is the frequency of the driving power. If *F*_0_ is the amplitude of the output force of the square piezoelectric vibrator, then assume that the vibration displacement and output force at time *t* are *X*_0_*(t)* and *F_0_*cos*wt* respectively, and *X(t)* is the vibration displacement of the pump chamber diaphragm. Thus, the motion differential equation of the system is

(1) $$\left\{ \begin{array}{l} M_{act}{\ddot{X}}_{0}+K_{act}X_{0}+K_{tra}\left(X_{0}-X\right)=F_{0}\cos\omega t\\ M_{dia}\ddot{X}+C\dot{X}-K_{tra}\left(X_{0}-X\right)+K_{dia}X=0 \end{array}\right..$$

In Equation (1), *F*_0_cos*ωt* is the output force from the center of the square piezoelectric vibrator. If the vibrator is considered as an ideal spring that provides vibration power, the mass of *M_act_* can be ignored, so the motion differential equation can be converted to

(2) $$M_{dia}\ddot{X}+C\dot{X}+\frac{K_{act}K_{tra}+K_{act}K_{dia}+K_{tra}K_{dia}}{K_{act}+K_{tra}}X=\frac{K_{tra}F_{0}\cos\omega t}{K_{act}+K_{tra}}.$$

The natural frequency of the system can be obtained from Equation (2):

(3) $$\omega_{n}=\sqrt{\frac{K_{act}K_{tra}+K_{act}K_{dia}+K_{act}K_{dia}}{M_{dia}\left(K_{act}+K_{tra}\right)}}.$$

$\zeta =\frac{C}{2M_{dia}\omega_{n}}$ is the damping ratio. The steady-state response amplitude of the system is obtained by using Laplace transform to Equation (2):

(4) $$X=\frac{K_{tra}}{K_{act}K_{tra}+K_{act}K_{dia}+K_{act}K_{dia}}\cdot \frac{F_{0}}{{\left[1-{\left(\frac{\omega }{\omega_{n}}\right)}^{2}\right]}^{2}+{\left(2\zeta \frac{\omega }{\omega_{n}}\right)}^{2}}.$$

The deformation under the static force *F*_0_ is

(5) $$\delta_{st}=\frac{K_{tra}F_{0}}{K_{act}K_{tra}+K_{act}K_{dia}+K_{act}K_{dia}}.$$

Then, the amplitude amplification coefficient of the system is calculated by Equations (4) and (5):

(6) $$\frac{X}{\delta_{st}}=\frac{1}{\sqrt{{\left[1-{\left(\frac{\omega }{\omega_{n}}\right)}^{2}\right]}^{2}+{\left(2\zeta \frac{\omega }{\omega_{n}}\right)}^{2}}}.$$

According to Equation (6), when the driving frequency fulfills $\omega =\omega_{n}$, the system resonates, with the amplitude amplification coefficient reaching maximum. Due to the damping of the vibration system, the amplification coefficient is limited.

Wheeled check valves with different structural parameters all have the feature of response hysteresis. The higher the driving frequency of the system, the more obvious the hysteresis, which will reduce the working efficiency of the wheeled check valve and lower the performance of the pump. Therefore, it is vitally important to select the appropriate system operating frequency to improve output performance. We can make a preliminary calculation on the resonance frequency, according to Equation (3). On this basis, considering the feature of hysteresis, the appropriate structural parameters for the wheeled check valve can be determined to improve the output performance.

## 4. Prototype Fabrication and Experimental Device

### 4.1. Experimental Prototypes

**Figure 5:**
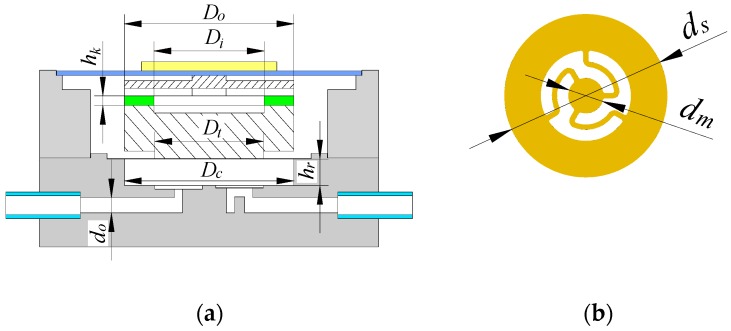
Key structural parameters of the piezoelectric resonance pump. (**a**) Main structure of the pump; (**b**) Structure of the wheeled check valve.

Figure 5a shows the key structural parameters of the piezoelectric resonance pump. *D_t_* is the diameter of the fixed connection part between the lower vibration transfer block and the pump chamber diaphragm, hereinafter referred to as “fixed connection diameter”. *D_c_* is the diameter of the pump chamber, *h_r_* is the height of the chamber and *d_o_* is the diameter of the flow channel. *D_i_* and *D_o_* are the inner and outer diameters of the annular elastic gasket in the middle of the elastic vibration transfer block, and *h_k_* is the thickness of the annular elastic gasket. Figure 5b shows the key structural parameters of the wheeled check valves at the inlet and outlet of the pump. *d_s_* is the outer diameter of the valve, *d_m_* is the outer diameter of the moving disc, and *k_v_* indicates the stiffness of the valve in the opening direction. These structural parameters, which have a direct impact on the output performance of the piezoelectric resonance pump, were tested experimentally in the following section. The main parameters for the prototype designed are shown below in Table 1.

**Figure 6:**
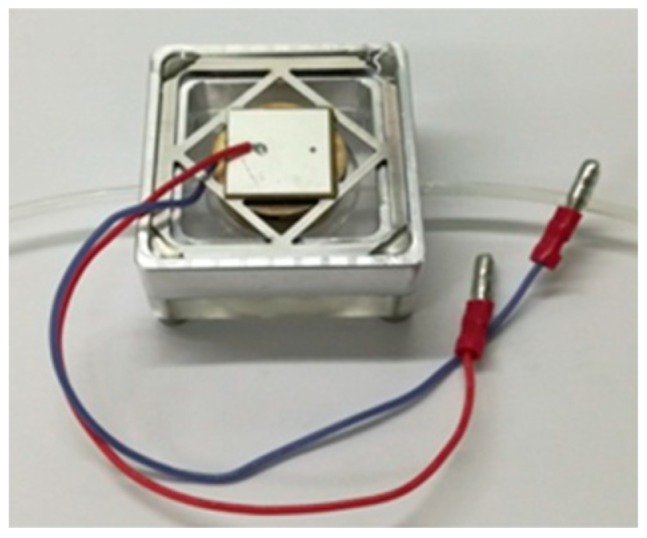
Photo of the piezoelectric resonance pump.

Figure 6 is a photo of the piezoelectric resonance pump designed in this paper. Its overall dimensions were 50 mm × 50 mm × 20 mm. The material of the pump body was polymethyl methacrylate (PMMA), which is highly transparent and convenient to observe the working status of the wheeled check valve. The diaphragm and the valve were made of beryllium bronze sheets, and the annular elastic gasket was made of 60Si2Mn. The 60Si2Mn is a kind of silicomanganese alloy spring steel and commonly used as the material for piezoelectric vibrator substrates. Beryllium bronze has features such as high strength, hardness, elastic limit and fatigue limit, and has a small elastic hysteresis.

### 4.2. Experimental Device

**Figure 7:**
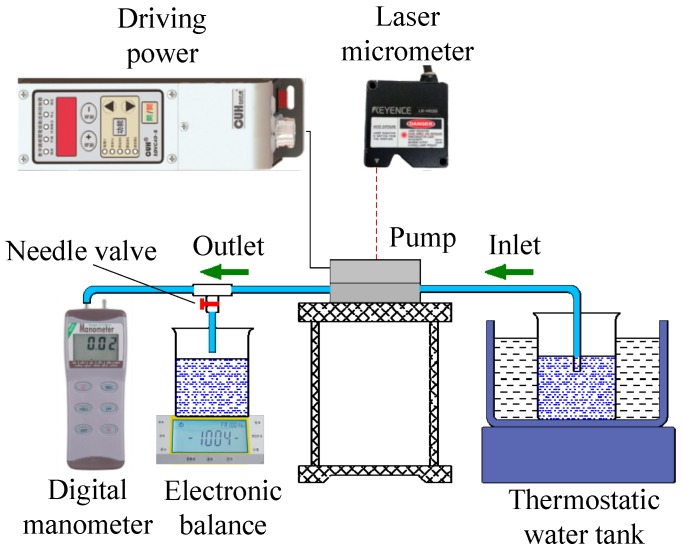
Schematic of the experimental device.

**Figure 8:**
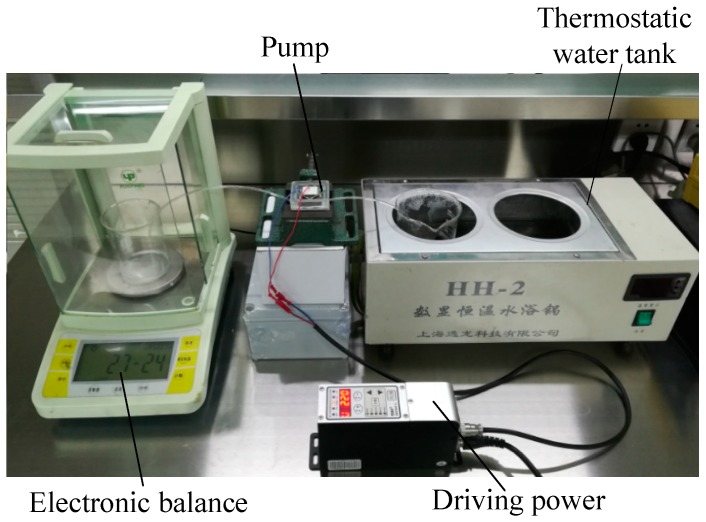
Photograph of the experimental device.

Figure 7 shows the performance testing device for the piezoelectric resonance pump. The SDVC-40 driving power source could generate sine wave driving signals, with a peak-to-peak voltage ranging from 0 to 220 V and a driving frequency ranging from 40 to 400 Hz. Water, as the pumped medium, was heated to 60 °C and the temperature was kept constant by a thermostatic water bath. The inlet and outlet tubes were placed horizontally on the bench, so the back pressure of the pump was zero. The flow rate of the piezoelectric resonance pump was measured by the weighing method, and the output pressure was measured by the digital manometer. The laser micrometer was used to measure the amplitudes of the piezoelectric vibrator and the diaphragm. Figure 8 is a photograph of the experimental device of the piezoelectric resonance pump. In order to lower the measurement error of the experimental data, each prototype was measured four times and the average value calculated.

## 5. Results and Analysis

### 5.1. Experiments on Amplitude–Frequency Characteristics

**Figure 9:**
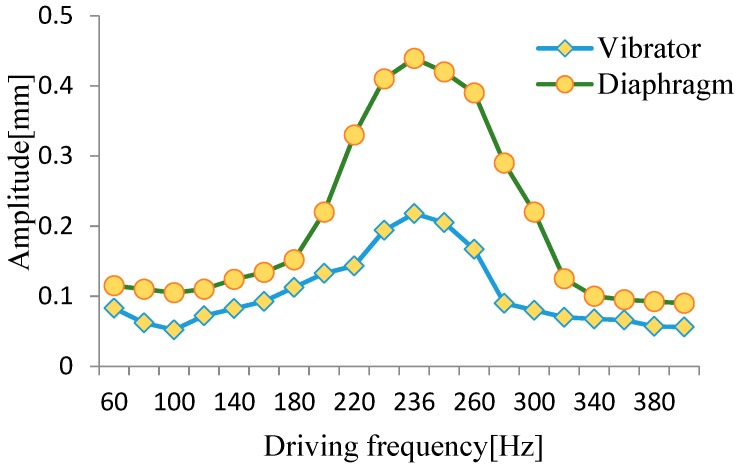
Amplitude–frequency characteristics of the pump vibration system.

Experimental prototypes were made for the performance tests. The amplitude–frequency characteristics of the pump vibration system were studied without water pumping. To begin with, we set the peak-to-peak voltage of the driving power as 220 V. Then we changed the driving frequency and measured the amplitude of the square piezoelectric vibrator and the pump chamber diaphragm by laser micrometer. The test results are shown in Figure 9; as the driving frequency increased, the amplitudes of the vibrator and the diaphragm both initially increased, but then declined. When the driving frequency was 236 Hz, the amplitudes of the vibrator and the diaphragm reached the maximum values of 0.21808 mm and 0.43933 mm, respectively. At this point, the system resonated.

### 5.2. Optimization Test of the Fixed Connection Diameter

**Figure 10:**
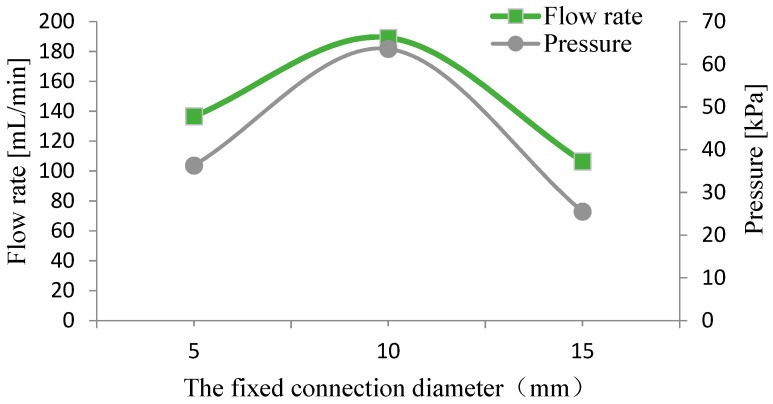
Output performance under different fixed connection diameters.

When the diameter of the pump chamber diaphragm was constant, the fixed connection diameter (*D_t_*) between the diaphragm and the transfer block can make a difference in the pump’s output capability. We chose the fixed connection diameter *D_t_* as 5 mm, 10 mm and 15 mm, respectively, to make prototypes to carry out three tests. The test results of the output flow rate and output pressure were recorded, as shown in Figure 10. The results showed that the piezoelectric resonance pump had the best output performance when the fixed connection diameter was 10 mm, with a flow rate of 189 mL/min and an output pressure of 63 kPa at this point.

### 5.3. Optimization Test of the Pump Chamber Height

Another important parameter of the pump is chamber height. If the chamber is too high, it will reduce the liquid compression ratio, while being too low will increase the flow resistance of the pump chamber. Therefore, we made five prototypes with different chamber heights to conduct tests, with the heights 0.15 mm, 0.20 mm, 0.25 mm, 0.30 mm and 0.35 mm. First, we opened the needle valve and tested the output flow rate of the prototypes without load. It was found that the resonance frequencies of the five prototypes differed little, ranging from 190 Hz to 213 Hz. When closing the needle valve and testing the output pressure, the resonance frequencies of the five prototypes also differed little, within the range of 170 Hz to 186 Hz. However, this was quite different from the situation when the prototypes were unloaded. The reason for this phenomenon is that the working load affects the stiffness and damping of the vibration system, thereby changing its resonance frequency.

**Figure 11:**
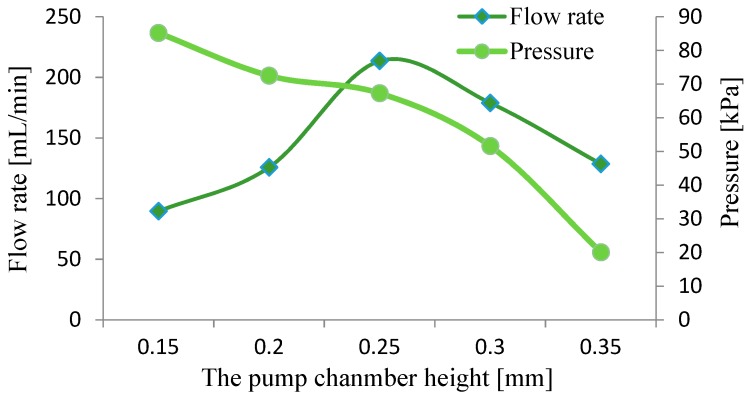
Output performance under different chamber heights.

The test results of the five prototypes with different chamber heights are shown in Figure 11. Comparing the trends between curves in the chart, it can be seen that the flow rate of the resonance pump first increased and then fell with the increase of the pump chamber height. When the height was 0.25 mm, the pump’s flow rate reached a maximum of 213.5 mL/min. Conversely, the output pressure of the resonance pump decreased as the chamber height increased, achieving the maximum of 85.2 kPa with a height of 0.15 mm. Increasing the chamber height meant lowering the liquid compression ratio in the pump chamber, and thus the output pressure was reduced.

### 5.4. Optimization Test of the Wheeled Check Valve

The response of the wheeled check valve has hysteresis, and the key factors affecting this hysteresis include the working frequency of the pump and the stiffness of the check valve itself. For a piezoelectric resonance pump working at a specific resonance frequency, it is essential to select a wheeled check valve with appropriate stiffness to improve the pump’s output performance.

**Figure 12:**
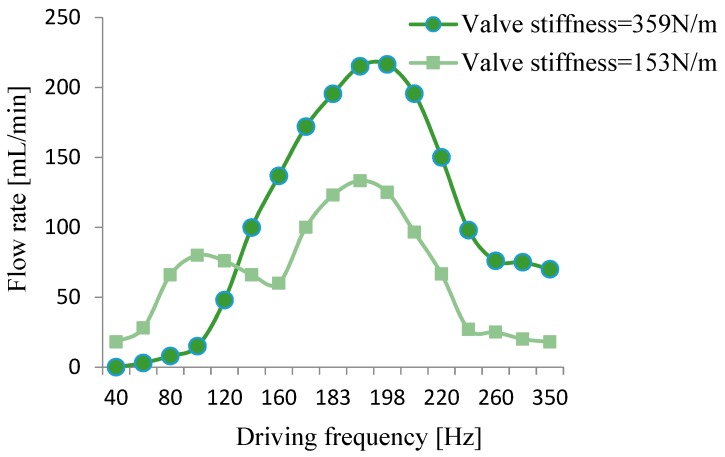
Relation curve between flow rate and valve stiffness.

**Figure 13:**
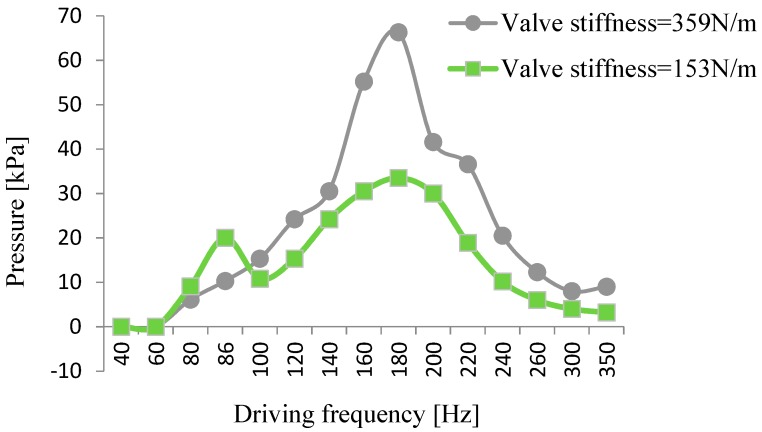
Relation curve between pressure and valve stiffness.

We used wheeled check valves with different stiffnesses to make prototypes, and found the appropriate stiffness by testing. Two valves, with a stiffness of 153 N/m and 359 N/m, were chosen for comparison, and the results are shown in Figure 12 and Figure 13. For the prototype with the valve stiffness of 153 N/m, the output flow rate and output pressure changed with the driving frequency. Two peaks were formed, one near the optimal working frequency of the wheeled check valve and the other near the resonance frequency of the pump. For the other prototype of 359 N/m, the flow rate and output pressure also changed with the driving frequency, but only one peak appeared near the pump’s resonance frequency. The above tests show that selecting a wheeled check valve with appropriate stiffness can improve the output performance of the piezoelectric resonance pump.

## 6. Conclusions

This paper discussed a piezoelectric resonance pump with a flexible support, which took a square piezoelectric vibrator as the driving unit. After analyzing the working principle and establishing a dynamic model of the vibration system, prototypes with different structural parameters were made and tested. Finally, the experimental results were provided to show several performance indexes to optimize the pump system.

A vibration test of the piezoelectric resonance pump was carried out. The results show that the pump resonated at 236 Hz when pumping air. When the peak-to-peak voltage of the driving power was 220 V, the amplitude of the diaphragm reached a maximum value of 0.43933 mm, and the volume change of the pump improved. This suggests that the flexible supported square piezoelectric vibrator can effectively reduce the constraints of fixed installation with its own vibration.

For the results of the performance test of the piezoelectric resonance pump show that when the pump chamber height was 0.25 mm, the output flow rate reached the maximum value of 213.5 mL/min. When the chamber height was 0.15 mm, the output pressure reached the maximum value of 85.2 kPa. This indicates that the chamber height directly affected the liquid compression ratio in the pump chamber. Increasing the chamber height means that the liquid compression ratio decreased, thereby reducing the output pressure. 

Finally, the check valve comparison test showed that it is vital to select a wheeled check valve with suitable stiffness according to the frequency for a piezoelectric resonance pump working at a specific resonance frequency. An appropriate check valve can improve the pump’s output performance.

## Figures and Tables

**Table 1:** The main structural parameters of the prototype.

Structural Parameters	Values
Fixed connection diameter *D_t_* (mm)	5, 10, 15
Diameter of the pump chamber *D_C_* (mm)	25
Height of the pump chamber *h_r_* (mm)	0.15, 0.2, 0.25, 0.3, 0.35
Diameter of the flow channel *d_o_* (mm)	2.1
Inner diameter of the annular elastic gasket *D_i_* (mm)	17
Outer diameter of the annular elastic gasket *D_o_* (mm)	25
Thickness of the annular elastic gasket *h_k_* (mm)	0.3
Outer diameter of the wheeled check valve *d_s_* (mm)	7
Outer diameter of the moving disc *d_m_* (mm)	1.5
